# Preventive medication use among persons with limited life expectancy

**DOI:** 10.1179/174329111X576698

**Published:** 2011-01

**Authors:** André R Maddison, Judith Fisher, Grace Johnston

**Affiliations:** 1 Faculty of Medicine, Dalhousie University, Halifax, Nova Scotia, Canada; 2 College of Pharmacy, Dalhousie University, Halifax, Nova Scotia, Canada; 3 School of Health Administration, Dalhousie University, Halifax, Nova Scotia, Canada

**Keywords:** Preventive medication use, Appropriate medication use, Limited life expectancy, Palliative care, Terminal care

## Abstract

Persons with limited life expectancy (LLE) – less than 1 year – are significant consumers of health care, are at increased risk of polypharmacy and adverse drug events, and have dynamic health statuses. Therefore, medication use among this population must be appropriate and regularly evaluated. The objective of this review is to assess the current state of knowledge and clinical practice presented in the literature regarding preventive medication use among persons with LLE. We searched Medline, Embase, and CINAHL using Medical Subject Headings. Broad searches were first conducted using the terms ‘terminal care or therapy’ or ‘advanced disease’ and ‘polypharmacy’ or ‘inappropriate medication’ or ‘preventive medicine’, followed by more specific searches using the terms ‘statins’ or ‘anti-hypertensives’ or ‘bisphosphonates’ or ‘laxatives’ and ‘terminal care’. Frameworks to assess appropriate versus inappropriate medications for persons with LLE, and the prevalence of potentially inappropriate medication use among this population, are presented. A considerable proportion of individuals with a known terminal condition continue to take chronic disease preventive medications until death despite questionable benefit. The addition of palliative preventive medications is advised. There is an indication that as death approaches the shift from a curative to palliative goal of care translates into a shift in medication use. This literature review is a first step towards improving medication use and decreasing polypharmacy in persons at the end of life. There is a need to develop consensus criteria to assess appropriate versus inappropriate medication use, specifically for individuals at the end of life.

## Introduction

Persons with limited life expectancy (LLE) – less than 1 year – are at increased risk of polypharmacy and adverse drug events, have complex and dynamic health statuses, and have unique health-care needs due to their LLE.^1,2^ Therefore, it is imperative that medication use among this population be appropriate and regularly evaluated.

Persons with LLE have an increased risk of polypharmacy, i.e. taking five or more medications, because they are commonly receiving medications to control their terminal disease and to manage pain and symptoms, as well as receiving medications for long-term prevention and management of chronic conditions.^3^ Polypharmacy is associated with a greater risk of adverse drug events because of drug–drug interactions and drug–disease interactions.^3–5^

The objective of this paper is to report on the current knowledge from the literature regarding preventive medication use among persons with LLE. This review begins by examining criteria and frameworks for assessing appropriate versus inappropriate medication use among persons with LLE. The literature that examines appropriate versus inappropriate medication use among this population is then presented. Lastly, we highlight the importance of evaluating the continuance in the use of long-term chronic disease preventive medications and the addition of palliative preventive medications for persons with LLE.

For the purpose of this paper, preventive medications are defined as drugs that are used to proactively manage a disease or symptom. There is an implicit assumption, which is not fully achievable in practice, that it is possible to prognosticate that a person has an LLE, and therefore adjust medication use accordingly.^6^ This paper is not a full systematic literature review, but rather is bounded to raise awareness and frame the issues. A comprehensive review of the extensive literature on palliative medication use is beyond the scope of this article. This literature review provides a useful and substantive base for future investigation into the use of preventive medications at end of life.

## Methods

A Medline, Embase, and Cumulative Index to Nursing and Allied Health Literature (CINAHL) search using the terms ‘preventive medication’ and ‘terminal care’ or ‘palliative care’ retrieved no articles. Therefore, we expanded the search to include the terms ‘terminal care or therapy’ or ‘palliative care or therapy’ or ‘advanced disease’ and ‘polypharmacy’ or ‘inappropriate medication’ or ‘preventive medicine’ and retrieved 341 papers. Most studies of medication use for persons with LLE focus on a specific medication, rather than preventive medications in general, or a specific outcome rather than a spectrum of events at end of life. Therefore, we conducted an additional search using the terms ‘statins’ or ‘anti-hypertensives’ or ‘bisphosphonates’ or ‘laxatives’ and ‘terminal care’ and retrieved an additional 23 articles. We then applied ISI Web of Knowledge's ‘cited reference’ facility to all included articles and hand-searched reference lists and identified a further 6 studies, giving a total of 370 potentially relevant papers.

**Figure 1:**
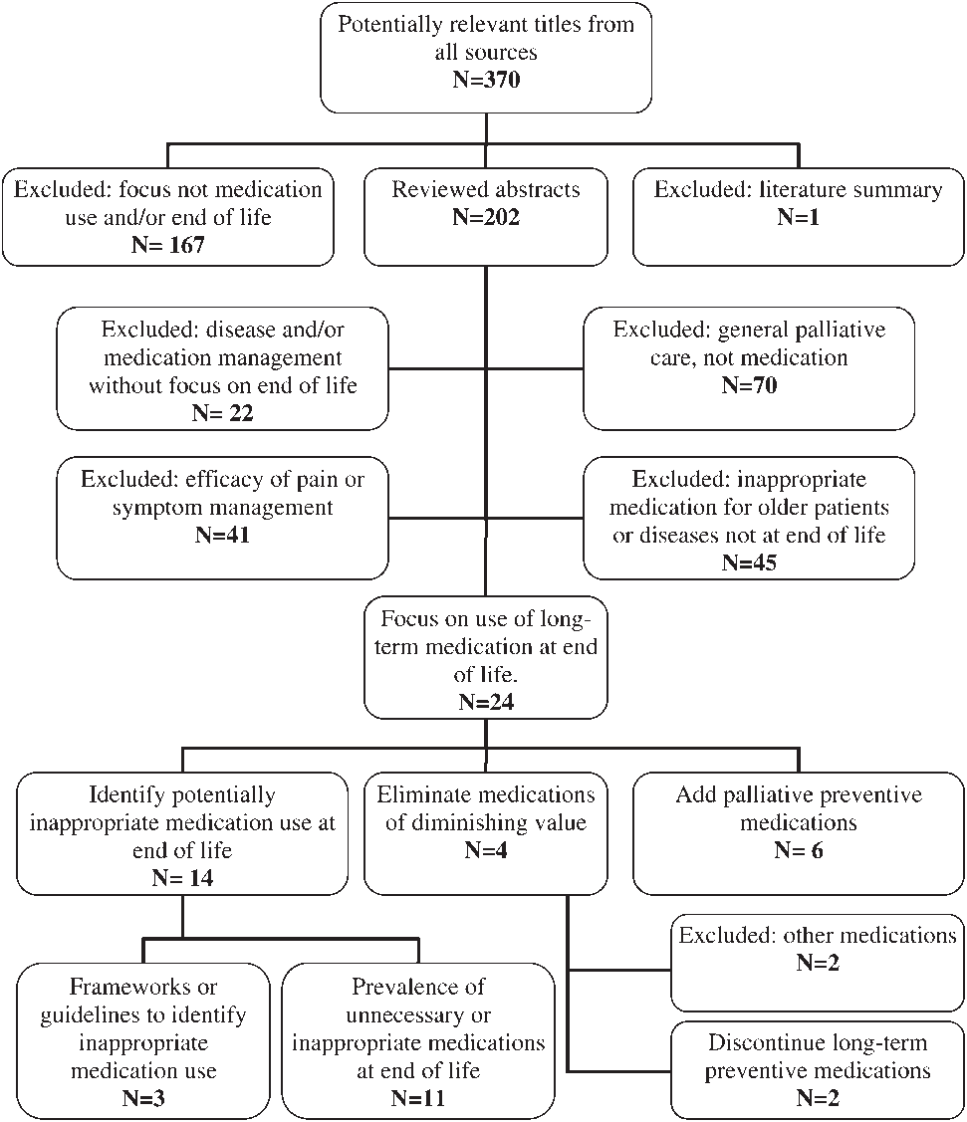
Process to identify relevant literature.

Each search was limited to studies published in English and was unrestricted by year of publication. Based on the abstracts, two of the authors (AM, JF) independently reviewed the citations and discussed differences on inclusion of articles into this review of appropriate versus inappropriate use of preventive medications among persons with LLE. The literature search process is summarized in Fig. 1.

## Results

### Frameworks and guidelines to identify appropriate versus inappropriate medications

Several criteria or indices have been developed to classify appropriate versus inappropriate medications for the elderly including the Beers criteria (or Beers list), the Medication Appropriateness Index, and the Screening Tool of Older Persons' Potentially Inappropriate Prescriptions (STOPP) criteria.^7–9^ A limitation of the Beers criteria and these other frameworks is that they are developed specifically for elderly populations, who are only a subset of persons at end of life.^2^ Individuals at the end of life are not all elderly and have varied and dynamic health conditions that are difficult to classify into a simple, yet comprehensive, medication appropriateness index or criterion. A diagnosis of LLE modifies the goals of care towards a more palliative and supportive focus, and subsequently alters the medications that are appropriate or inappropriate.^10^

The literature search identified three papers that address the issue of developing specific frameworks or guidelines for identifying appropriate versus inappropriate medication use among persons with LLE.^2,10,11^ Of these three articles, two (Holmes *et al.* and Bain and Weschules) present specific guidelines or frameworks for assessing appropriate medication use at end of life. The remaining paper^11^ is a letter to the editor that highlights the need to address this issue.

Bain and Weschules^10^ examined the feasibility of applying the Beers criteria to medication use among older adults receiving hospice care. They developed a list of potentially inappropriate medications based on the Beers criteria and The Hospice Pharmacia Medication Use Guidelines™ seventh edition. The latter is a set of proprietary pharmacotherapy-based guidelines and algorithms specific to hospice care designed as a clinical tool for the pharmacological management of symptoms common at end of life.^12^ Twenty-four members of a multidisciplinary panel reviewed the list and assessed the appropriateness of each medication or medication class for hospice patients aged 65 and older. The panelists considered some medications, e.g. propoxyphene, to be inappropriate for all patients; however, some medications, e.g. lorazepam and haloperidol, which were otherwise considered inappropriate according to the Beers criteria, were judged to be appropriate for hospice care. The authors note the need to develop potentially inappropriate medication criteria that are specific to hospice care.

Holmes and colleagues designed a medications model that is specific to persons with LLE.^2^ This model incorporates individuals' remaining life expectancy, goals of care, and time until benefit of medications.^2^ The Holmes *et al.* prescribing model is less explicit than the Beers criteria and is meant to assist health-care providers in making decisions regarding medication use for persons at the end of life. The Holmes *et al.* prescribing model does not provide specific guidelines regarding appropriate versus inappropriate medications, but does provide a framework to direct prescribing to avoid inappropriate or ineffective medication use.

### Prevalence of potentially inappropriate medication use at end of life

Although there is literature examining potentially inappropriate use of medications among the elderly,^13–17^ few studies focus specifically on preventive medications and persons with LLE. Our search retrieved only 11 studies, summarized in Table 1, which focus on the appropriateness of medication use among persons with LLE.^17–27^

**Table 1:** Studies examining prevalence of unnecessary and inappropriate medication use among individuals with LLE

Study	Study location	Population	Outcomes	Criteria/index
Blass *et al.* (2008)^18^	Baltimore, Maryland, USA	125 nursing home residents with advanced dementia	Total number and type of prescribed medications at baseline, with changes prior to death	Medications were not assessed as appropriate versus inappropriate
		88 patients died within 6-month study period		
Currow *et al.* (2007)^19^	Adelaide, South Australia	260 patients referred to palliative care programs	Medication use from palliative care referral to death	Medications classified as either for co-morbid conditions or for symptom control using the Beers' consensus criteria
Fahlman *et al.* (2007)^17^	USA	4602 community elderly (>65) from a managed care organization	Use of potentially inappropriate medications during last year of life	Beers consensus criteria
Fede *et al.* (2010)^20^	Sao Paulo, Brazil	87 patients with advanced cancer	Use of unnecessary medications	Unnecessary medications determined by authors through a literature search and expert consensus
Holmes *et al.* (2008)^21^	Illinois, USA	34 community and long-term care residents with advanced dementia	Appropriate versus inappropriate medication used by individuals with advanced dementia	Appropriate versus inappropriate determined by panel of 12 geriatricians; classified as never, rarely, sometimes, or always appropriate, or no consensus
Koh and Koo (2002)^22^	Singapore	345 patients in an in-patient palliative care program, in-patient hospice program, or receiving home care service	Number of medications taken prior to and after a palliative care referral	Futility or appropriateness not assessed
Nicholson *et al.* (2001)^23^	England	106 patients admitted to St Benedict's Hospice	Number of futile or inappropriate medications when admitted	Futility and inappropriateness assessed by physician
Riechelmann *et al.* (2009)^24^	Toronto, ON, Canada	372 patients with advanced cancer receiving care in palliative care clinics	Proportion taking futile medications	Futility of medications determined by authors; classified as duplicates or unnecessary
Silveira *et al.* (2008)^25^	Central Illinois, Indiana, Michigan, and Northwest Ohio, USA	337 cases with LLE versus 1247 controls without LLE; LLE identified using Palliative Care Index diagnoses	Prevalence of statin use during last 6 months of life; variations by presence or absence of LLE	Futility or appropriateness not assessed
Suhrie *et al.* (2009)^26^	Pittsburgh, Pennsylvania, USA	89 patients who died in a geriatric palliative care unit	Unnecessary medication use at first admission and in last 30 days before death from pharmacist drug review	Assessed using unnecessary drug use measure, a classification of Medication Appropriateness Index
Tanvetyanon and Choudhury (2006)^27^	Chicago, Illinois, USA	47 patients with advanced lung cancer receiving statins at diagnosis	Discontinuation of statins by timing and characteristics of discontinuation	Futility or appropriateness not assessed

A considerable number of individuals with LLE continue to receive potentially inappropriate medications, yet results vary by study location and population. Fahlman *et al.*^17^ retrospectively applied the Beers criteria to examine the extent of inappropriate medication use among 4602 community-dwelling individuals in the last year of life in the USA. They identified that 44% of participants received at least one potentially inappropriate medication in their last year of life.^17^ In contrast, Nicholson *et al.*^23^ identified that 25% of individuals admitted to a hospice in England were taking futile or unnecessary medications according to the admitting physicians.

In order to assess the changes in potentially inappropriate medication use, Currow *et al.*^19^ examined medication use each month from palliative care referral until death. They identified that the total number of medications taken increased as death approached due to a greater number of symptom-specific medications, while medications for chronic diseases decreased. Moreover, two studies analyzed the impact of a palliative care consultation on medication use. Without distinguishing between appropriate and inappropriate medications, Koh and Koo^22^ identified that the total number of medications taken prior to and after palliative care assessments in Singapore remained unchanged. Suhrie *et al.*^26^ applied the Medication Appropriateness Index^8^ to examine medication use by 89 individuals before and after admission to a palliative care unit in Pittsburg, PA. For the purpose of their study, unnecessary medications were defined as those that lacked indication (e.g. prescribing iron for an individual without an iron deficiency), lacked effectiveness (e.g. prescribing a drug that has greater side effects than an alternative drug), or were duplicate medication. They identified that the use of unnecessary medications declined from a mean of 1.7 to 0.6 medications per person after admission to the palliative care unit.^26^

To more precisely report on the appropriateness of medication use, selected studies restricted their analysis to a specific condition or specific preventive medication. The literature search retrieved four studies that examined specific conditions: advanced dementia^18,21^ and terminal cancer.^20,24^ Holmes *et al.*^21^ examined medication use in 34 persons with advanced dementia who were registered in a palliative care program and assessed appropriateness using a Delphi panel of 13 geriatricians. Twenty-nine percent of persons were receiving a medication that was classified as never appropriate, including HMG-CoA (3-hydroxy-3-methyl-glutaryl coenzyme A) reductase inhibitors (statins), acetylcholinesterase inhibitors, estrogen, and clopidogrel, and 21% were receiving medication that were rarely appropriate.^21^ Blass *et al.*^18^ examined medication use among nursing home residents with advanced dementia (*n* = 125), in particular how medication use changed as death approached. While the total number of medications prescribed did not vary, the use of opioid analgesics increased, while the use of most other medications decreased, e.g. for cardiovascular disease and dementia.^18^ While the study did not determine the appropriateness of the medications, the high rates of medication use are of concern.

Riechelmann *et al.*^24^ retrospectively examined 372 persons with advanced/incurable malignancies who were registered with a palliative care program. The study identified that 22% of individuals were receiving at least one unnecessary or duplicate medication. Fifty-six percent of unnecessary medications were statins and 88% of duplicate medications were benzodiazepines.^24^ In comparison, Fede *et al.*^20^ reported that 24% of individuals with advanced cancer in Sao Paulo, Brazil, were receiving unnecessary medication according to the admitting physician.

Two studies (Silveira *et al.*^25^ and Tanvetyanon and Choudhury^27^) focused on statin use in persons with LLE. Silveira *et al.*^25^ used a matched case–control study design to examine whether individuals with a recognizable life-limiting condition would have statins discontinued more often and more quickly than a control group. They compared statin discontinuation among persons with a recognizable life-limiting condition, as defined by the Veterans Health Administration Palliative Care Diagnoses Index, to those who died of other causes, matched on age, sex, co-morbid conditions, and socioeconomic status. Silveira *et al.*^25^ identified that 51% of cases and 64% of controls received statins until death. There was no statistically significant difference in the timing of discontinuation between cases and controls. Tanvetyanon and Choudhury^27^ examined patterns of statin discontinuation among 47 persons who were diagnosed with stage IIIb or IV lung cancer and were taking statins at the time of diagnosis. Eighty-nine percent of persons received at least one prescription renewal after diagnosis. Statins were eventually discontinued in 53%. The median time of discontinuation was approximately 250 days after diagnosis.^27^

The 11 studies examining appropriate versus inappropriate medication use among persons with LLE suggest that a considerable proportion of individuals at the end of life continue to take chronic disease prevention medications and other potentially inappropriate or unnecessary medications. However, there is an indication that as death approaches the shift from curative to palliative goals of care translates into some change in medication use. Beyond these 11 studies that report on the prevalence of unnecessary and inappropriate medication at end of life, two additional papers focus on discontinuing preventive chronic disease medications, and a further six on adding palliative preventive medications.

### Eliminating chronic disease preventive medications of diminishing value

The literature search identified only two papers with a specific focus on discontinuing chronic disease preventive medications among persons with LLE.^1,3^ Individuals in the general population are prescribed long-term medications such as statins to control cholesterol levels, anti-hypertensives to control blood pressure, and bisphosphonates to maintain bone density. However, if a person is diagnosed with a terminal disease, the benefit of these long-term preventive medications may become negligible, and the potential for harm may outweigh the benefit.^28^ The safety, efficacy, and benefits of statins, anti-hypertensives, and bisphosphonates have been demonstrated for the general population through several randomized control trials;^29–31^ however, there is limited evidence of their appropriateness among persons with LLE, in part because persons with LLE are rarely included in clinical trials.^3^ There are changes in pharmacodynamics and pharmacokinetics caused by terminal diseases, which result in medications reacting differently than expected. This leads to potentially greater risk of adverse effects and possible reduction of benefit.^28^ Time until benefit – the period of time required for a medication to be of value to a person – of preventive medications must also be considered, as it may extend beyond life expectancy. Therefore, there is a need to evaluate prescribing for persons with LLE and to consider discontinuing medications that are no longer beneficial.^2^ The use of statins is a case in point.

Statins are prescribed for the primary prevention – prior to disease occurrence – and tertiary prevention – minimizing effects of a disease that is present and avoiding re-occurrence – of cardiovascular disease.^29–31^ If the goal of care is palliative, long-term prevention of cardiovascular disease is potentially unnecessary, may contribute to polypharmacy, and is an additional cost to the person and/or health system.^3^ Lastly, persons suffering from advanced disease usually have a natural decrease in cholesterol level through weight loss and studies have shown increased risk of adverse effects from statins among persons with LLE.^32,33^

In addition to the increased risk of polypharmacy and thus possible adverse events, preventive medication use by persons with LLE has psychological and financial considerations.^1^ If a preventive medication is discontinued in an individual with LLE, they may feel that the health-care providers are giving up hope. In contrast, renewing a person's preventive medication may provide false hope. Therefore, continuation or discontinuation of a preventive medication should be clearly discussed with the individual before being put into practice.^1^

**Figure 2:**
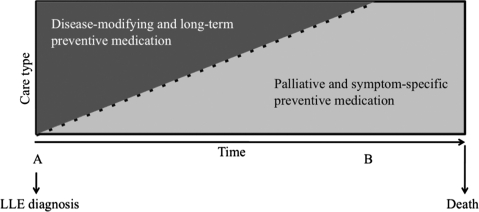
Conceptualizing the transition in medication use at end of life. (A) Goal of care begins to transition towards palliative and supportive care. (B) Goal of care is solely supportive and palliative. All long-term preventive medications and disease-modifying medications discontinued.

In summary, the use of chronic medications that reduce the risk of the occurrence or exacerbation of disease many years in the future should be reconsidered for persons whose life expectancy is limited. However, the introduction of new medications that prevent or reduce the risk of disease- or treatment-related adverse events may be indicated. This shift is illustrated in Fig. 2.

### Adding palliative preventive medications

The search strategy identified six papers that specifically address the issue of adding medications to the treatment regimen at end of life to avoid anticipated adverse outcomes.^34–39^ For example, it is known that bisphosphonates may prevent bone loss associated with androgen-deprivation therapy for prostate cancer and metastases to the bone from breast cancer.^34^ Beyond a disease-specific preventive focus, symptom-specific preventive medications can also be beneficial. The use of laxatives to counter the constipating effects of opioid painkillers is a case in point.^35^

Constipation is one of the most common symptoms experienced by persons with LLE.^36^ Constipation causes abdominal pain, cramping, and nausea and vomiting, and results in substantial declines in quality of life. The prevalence of constipation among patients receiving palliative care has been reported to be as high as 87%.^35^ Numerous factors contribute to the high prevalence of constipation among persons with LLE, in particular the use of opioids, dehydration, inactivity, difficulty eating, and specific disease-related effects.^35,36^ It is clinically recommended that laxatives be prescribed concurrently with a strong opioid in order to prevent opioid-induced constipation.^35–37^ Bouvy *et al.*^38^ examined concurrent laxative and strong opioid use among 269 elderly persons throughout the Netherlands. They identified that only 37% of these individuals were prescribed a laxative within 5 days of starting a strong opioid. However, Goodman *et al.*^39^ examined laxative use specifically among persons who were admitted to 1 of 11 hospices in the UK and found that 74% received laxatives with strong opioids.

## Recommendations and conclusion

Medication use should be regularly monitored and evaluated throughout a person's lifetime. However, when a person is diagnosed with an LLE condition, it is vitally important to reassess medication use to align with the goals of care and life expectancy. Although timing and medication use vary from person to person, the shift in goals of care as death approaches should be accompanied by ongoing evaluation of and appropriate changes in preventive medication use.

The literature examining the appropriate use of long-term preventive medications among persons with LLE contains several gaps, which should be addressed. Notably, there is minimal consensus in how best to assess medication use at end of life and varied definitions of inappropriate and futile medications. To date, there is limited research that reports whether ineffective or inappropriate preventive medications are being discontinued and whether appropriate palliative preventive medications are being initiated. Evidence of the discontinuation of potentially inappropriate preventive medication is limited mostly to descriptive or pilot studies and specific forms of potential appropriate/inappropriate use of preventive medications. In order to improve medication use, decrease polypharmacy and adverse drug events, reduce preventable adverse end-of-life events, and optimize medication costs, the first step is to develop a consensus framework or criterion to evaluate medication use at end of life, then to identify current patterns of use among persons with LLE. Inclusion of the key word(s) ‘preventive medication’ and ‘terminal care’ in papers on medications for persons with LLE would enable future literature searches.

In the meantime, health-care providers can promote optimal medication use among persons with LLE by regularly and critically evaluating the medication regimens of their patients within this population to assess the appropriateness of the continued use of preventive medications. The Holmes *et al.*^2^ prescribing model may provide a useful framework for this evaluation. Further, decisions to initiate, continue, or discontinue preventive medications at end of life should be preceded by meaningful dialogue and communication with the person with LLE.
